# Dental Bleaching with Phthalocyanine Photosensitizers: Effects on Dentin Color and Collagen Content

**DOI:** 10.3390/molecules28104223

**Published:** 2023-05-22

**Authors:** Zhouyan Wu, Guodong Wang, Zhiming Li, Zhengquan Li, Dandan Huang, Mingdong Huang, Minkui Lin

**Affiliations:** 1 Fujian Key Laboratory of Oral Diseases & Fujian Provincial Engineering Research Center of Oral Biomaterial & Stomatological Key Lab of Fujian College and University, School and Hospital of Stomatology, Fujian Medical University, 246 Yangqiao Zhong Road, Fuzhou 350002, China; 15659036770@163.com (Z.W.);; 2 The Second Affiliated Hospital of Fujian University of Traditional Chinese Medicine, 282 Wusi Road, Fuzhou 350003, China; 3 College of Chemistry, Fuzhou University, 2 Xueyuan Road, Fuzhou 350108, China; 4 Institute of Stomatology & Laboratory of Oral Tissue Engineering, School and Hospital of Stomatology, Fujian Medical University, Fuzhou 350002, China

**Keywords:** photodynamic therapy, hydrogen peroxide, tooth staining, Orange II, tooth bleaching, collagen type I

## Abstract

With the increasing demand for tooth bleaching in esthetic dentistry, its safety has been the focus of a comprehensive body of literature. In this context, the aim of the present study was to evaluate the application effects of pentalysine β-carbonylphthalocyanine zinc (ZnPc(Lys)_5_)-mediated photodynamic therapy in dentin bleaching and its effects on dentin collagen. We first established a new and reproducible tooth staining model using dentin blocks stained by Orange II and then bleached with ZnPc(Lys)_5_ (25 μM) and hydrogen peroxide (10% or 30%). Data were analyzed with one- and two-way ANOVA and a significance level of *p* < 0.05. ZnPc(Lys)_5_ effectively bleached the dentin samples to an extent comparable to hydrogen peroxide at either 10% or 30% concentrations. Further studies on the dentin morphology, chemical element distribution, and protein constituents, using an electron microscope, energy dispersive spectroscopy, X-ray photoelectron spectroscopy, and SDS-PAGE, demonstrated that treatment with the photosensitizer preserved the dentin structure and, at the same time, the major organic component, collagen type I. For comparison, hydrogen peroxide (10% or 30%) treatment significantly degraded the collagen protein. This work indicated that the photosensitizer exerts potent bleaching effects on dentin staining; importantly, does not damage dentin and its collagen content; and opens up a new strategy to further explore various photosensitizers for the bleaching of both tooth enamel and dentin.

## 1. Introduction

Tooth whitening is a critical component for an attractive smile and is useful in social interaction. In dental practice, tooth color is essential in oral examination, diagnosis and treatment [1]. The causes of tooth discoloration are varied and can be categorized into extrinsic, intrinsic, and internalized discoloration [2]. Extrinsic discoloration is defined as the staining of the tooth surface or the acquired enamel pellicle. Factors affecting extrinsic discoloration include age, inadequate brushing, smoking, the consumption of colored foods, and exposure to iron salt and chlorhexidine [3,4,5]. As this staining occurs on the surface of the teeth, it does not cause color changes in the internal structure. Conversely, intrinsic discoloration refers to the staining of internal tissues of the teeth caused by local factors, drugs, or systemic causes [2]. In addition, some researchers have proposed a third type of staining known as internalized discoloration, in which exogenous pigment molecules infiltrate into the tooth through the porous structure of a tooth defect, thus causing the staining of the internal tissue of the tooth [5,6]. Extrinsic discoloration can be treated by various methods including routine prevention, polishing, and sandblasting. For intrinsic discoloration, the treatment options include bleaching, resin infiltration, and crown or veneer restoration [7,8]. Compared to crown or veneer restoration, tooth bleaching is more conservative and less risky and, therefore, is the favored option.

Currently, hydrogen peroxide (HP) is the effective component of bleaching materials commonly used in clinical settings. HP can be used directly on teeth or indirectly generated by the decomposition of carbamide peroxide or sodium perborate to form HP, which then acts on teeth. The free radicals produced by HP decomposition can infiltrate into the tooth [9,10] and eventually lead to tooth bleaching [11]. Nonetheless, the use of HP bleaching products may be accompanied by side effects, such as tooth sensitivity, gingival irritation, tooth acid erosion and demineralization, and pulp damage [12,13]. One severe complication of tooth bleaching is external cervical resorption, and its incidence varies greatly, ranging from 1% to 13% [14,15,16,17]. Mature dentin is composed of 70% minerals, 20% organic matter, and 10% water by weight. The minerals in dentin are composed of hydroxyapatite, and collagen type I is the main component of the organic matter, accounting for 90% of the weight of organic components [18]. The hydroxyl free radical produced by HP can activate matrix metalloproteinases to degrade collagen in dentin and thus reduce the content of tooth organic matter [19,20,21,22,23]. Accordingly, teeth become more brittle and can fracture more easily. Our current study identifies a bleaching material that can not only decrease the side effects of tooth bleaching but also achieve a good tooth bleaching effect.

Photodynamic therapy (PDT) has been approved and used for the treatment of tumors [24,25]. In recent years, photodynamic therapy has been used in the treatment of oral diseases, including periodontitis, endodontics, precancerous lesions, and oral mucosal diseases [26,27,28,29,30]. Zhang et al. [31] developed a photosensitizer that could whiten teeth without damage. Gao et al. [32] found a similar result. Our previous studies demonstrated the bleaching effects of a new photosensitizer, pentalysine β-carbonylphthalocyanine zinc (ZnPc(Lys)_5_), in tooth bleaching and its effects on the morphology, roughness, and hardness of the enamel surface [33]. However, the role of photodynamic therapy in dentin bleaching and its effects on dentin have yet to be evaluated. Here, we investigate the application effects of ZnPc(Lys)_5_-mediated photodynamic therapy in dentin bleaching and its effects on dentin collagen.

We also developed a new tooth staining model. Staining materials such as blood, tea, and coffee are often used to develop in vitro tooth staining models [34,35,36]. Sulieman et al. [37] used tea to develop a model of dental staining to standardize in vitro research. Nevertheless, since the components of tea and coffee that cause tooth staining cannot be quantified, there may be differences among different brands and processes of tea and coffee. It would not be easy for researchers in different countries to obtain the same tea to reproduce the model. Orange II is a small molecular dye with a clear chemical structure and easy commercial availability. Orange II is a phenolic compound with some similarity to the phenol chromogens found in tea and coffee [38]. We demonstrate that Orange II can easily penetrate teeth to cause tooth staining, leading to a reproducible staining model.

Herein, we applied ZnPc(Lys)_5_-mediated photodynamic therapy to an Orange II solution to evaluate the bleaching effect of photosensitizers. A dentin staining model based on Orange II was established in vitro and used to study the tooth bleaching ability of ZnPc(Lys)_5_ by comparing it to hydrogen peroxide (10% and 30%). We repeatedly stained the bleached dentin blocks, obtained from a clinic, with Orange II. The damage to collagen I protein, the main organic component of dentin, was indirectly accessed by comparing the recolor ability of dentin blocks. Scanning electron microscopy (SEM) was used to study dentin surface morphology. The dentin content was analyzed by energy dispersive spectroscopy (EDS) and X-ray photoelectron spectroscopy (XPS). The purified human collagen type I was also treated directly with those tooth bleaching agents and then characterized by sodium dodecyl sulfate polyacrylamide gel electrophoresis (SDS–PAGE). Dynamic light scattering (DLS) was used to evaluate the changes in collagen particle size after bleaching to further study the effects of ZnPc(Lys)_5_ on collagen I. ZnPc(Lys)_5_-mediated photodynamic therapy showed potent bleaching efficacy without damaging collagen. This study presents a promising strategy of PDT for tooth dentin bleaching.

## 2. Results and Discussion

### 2.1. The Degradation of Orange II In Vitro by PDT

**Figure 1:**
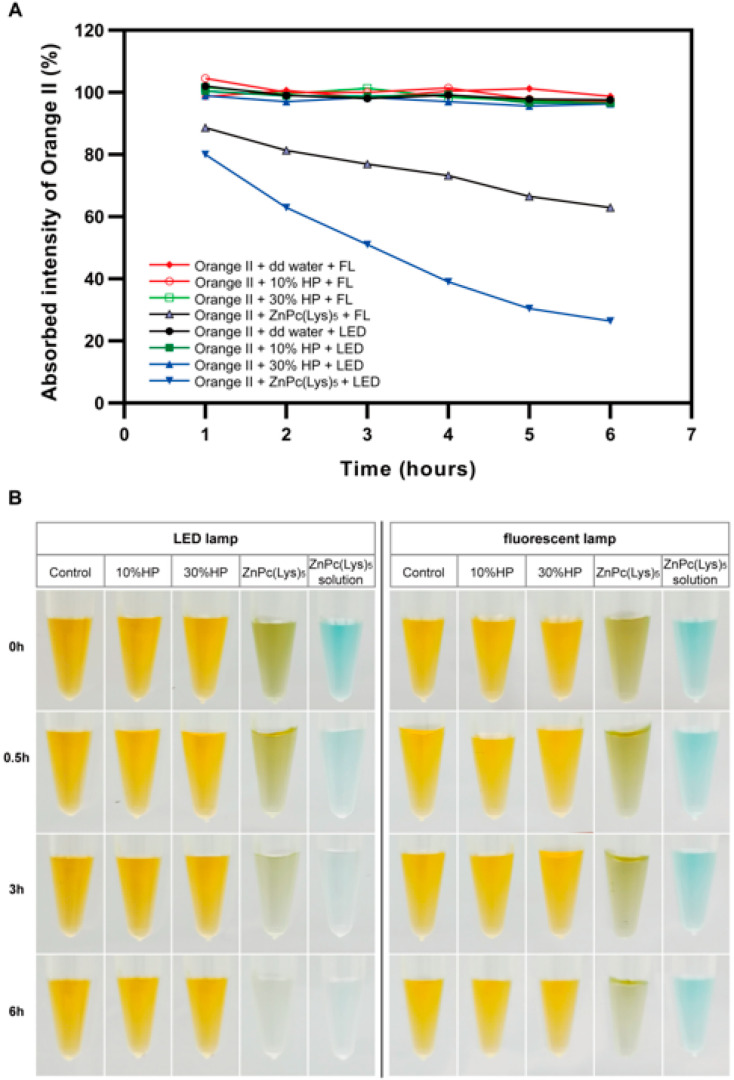
Discoloration of Orange II by ZnPc(Lys)_5_ photosensitizer and HP in solution. (**A**) Absorbed intensity of Orange II with deionized water, 10%HP, 30%HP, and ZnPc(Lys)_5_ at various reaction times. FL: Fluorescent lamp; dd water: deionized water. (**B**) Photographs of the Eppendorf tubes with different solutions at various reaction times.

To evaluate the bleaching effects of ZnPc(Lys)_5_, we first compared the degradation effects of different bleaching agents on Orange II in vitro. Due to the positive correlation between the Orange II concentration and the absorption peak, the concentration of Orange II can be estimated by using an enzyme labeling instrument to detect the absorption peak of Orange II at a specific wavelength (484 nm). Figure 1A shows the degradation of Orange II after mixing with different bleaching solutions at different time points by a microplate reader. It shows that the absorbance of Orange II decreased with time after mixing with ZnPc(Lys)_5_ and exposure to a light emitting diode (LED) lamp or a fluorescent lamp for 6 h. Notably, the absorbance value decreased more significantly under the action of the LED lamp, indicating that Orange II faded more obviously. Nevertheless, the absorbance of Orange II in the deionized water group, the 10% HP group, and the 30% HP group did not decrease significantly regardless of the LED lamp or the fluorescent lamp. This result is similar to the research results of Lee et al. [38], who showed that the concentration of Orange II decreased by about 1% after 30% HP reacted with the same volume of Orange II for 60 min. The reason for the low degradation of Orange II may be that when HP is mixed with the same volume of Orange II, the concentration of HP decreased by half, therefore resulting in a slower degradation rate of Orange II. It is worth noting that, compared to the degradation rate of Orange II with 10% HP and 30% HP, we found that ZnPc(Lys)_5_ degraded Orange II more quickly after 6 h, indicating that ZnPc(Lys)_5_ as a bleaching agent can significantly increase the degradation rate of Orange II and that the degradation rate of ZnPc(Lys)_5_ on Orange II is significantly higher than that of 10% HP and 30% HP. On the other hand, the photosensitizer consumption can be slow under ideal conditions. It can react with Orange II continuously under specific wavelength light, while HP will be continuously consumed after redox reaction with Orange II with the increase in reaction time, resulting in the decrease in the consumption rate of Orange II by HP [39]. There is a certain degree of ZnPc(Lys)_5_ photobleaching in this experiment, which shows that the original solution of ZnPc(Lys)_5_ becomes lighter with the extension of illumination time (Figure 1B). However, from the experimental results, we can infer that Orange II has been completely decomposed before the complete photobleaching of photosensitizer.

To evaluate the color change in Orange II more directly, we took pictures at 0, 0.5, 3, and 6 h. As shown in Figure 1B, Orange II mixed with ZnPc(Lys)_5_ became colorless after 6 h. In contrast, it did not show significant discoloration in deionized water, 10% HP, and 30% HP. Notably, ZnPc(Lys)_5_ could completely fade the color of Orange II after 6 h, and the color of ZnPc(Lys)_5_ itself was bleached as well under the irradiation of the LED lamp. However, the color of Orange II changed a little upon the treatment of ZnPc(Lys)_5_ for 6 h, and the photobleaching effects of ZnPc(Lys)_5_ were not noticeable under a fluorescent lamp.

### 2.2. Development of Orange II-Stained Tooth Staining Model

**Figure 2:**
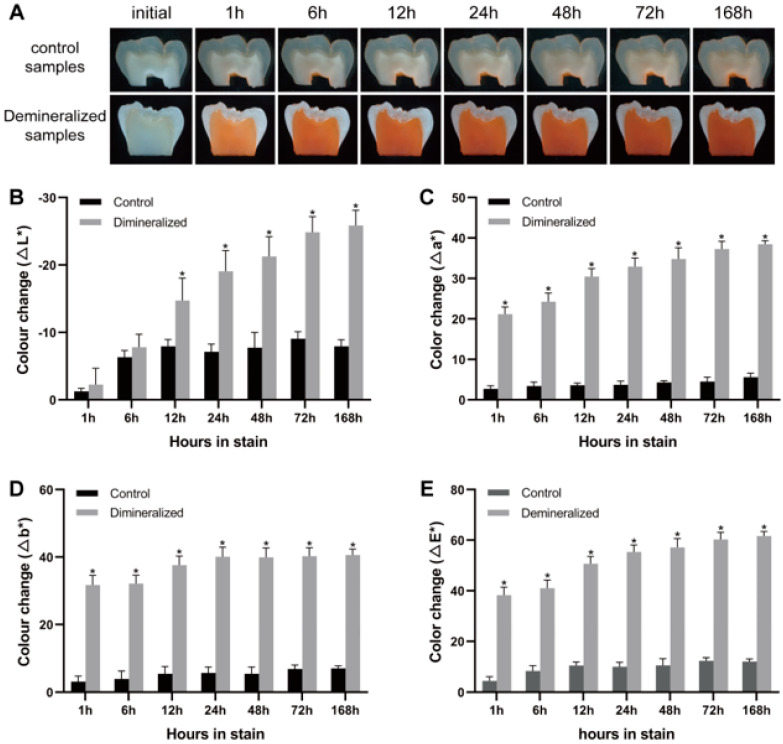
Color changes in the staining samples. (**A**) The color changes of demineralized and control dentin samples after staining with Orange II for 1−168 h. (**B**−**E**) ∆L*, ∆a*, ∆b*, and mean color change (∆E*) of dentin samples stained by Orange II for 1−168 h. Significant differences are indicated by * compared to the control group at different time points.

We used Orange II to stain dentin blocks and compared the effects of demineralized and non-demineralized treatments on the staining intensity of dentin blocks. The staining process of dentin blocks by Orange II was recorded using a stereoscopic microscope (Figure 2A). The L*a*b* values of each dentin block before and after dentine staining were measured by an image processing software, and the values of ∆L*, ∆a*, ∆b*, and ∆E* were calculated to quantitatively analyze the changes in dentine staining intensity. Both the demineralized samples and the control samples were stained with Orange II after staining for 168 h, and the demineralized samples absorbed more stains compared to the control samples (*p* < 0.0001 for all analyzed time points). Our findings indicated that the staining degree of dentin samples in the demineralized group was significantly enhanced compared to the control group, while the staining intensity of the control group did not change significantly (Figure 2B–E). The staining results of dentin blocks in the demineralization group are consistent with those of Alraies [40]. Their research revealed that demineralization treatment could enhance the staining degree of dentin blocks. However, the staining results of dentin blocks in the untreated control group were different from those in the previous studies. Previous studies showed that the non-demineralized dentin blocks could be clearly stained within a few days [38,40,41], while in our study, the untreated intact dentin blocks were not stained significantly. This difference could be because the treatment method of the dentin block in this study is different from prior methods. Additionally, this study adds the step of polishing with 600–1200 grit silicon carbide paper, which reduces the roughness of the dentin surface and the deposition of pigment on the tooth surface [42] to standardize the staining degree of the sample.

### 2.3. Stained-Tooth Bleaching Experiments

We have previously reported that ZnPc(Lys)_5_ had a significant bleaching effect on Orange II solutions. However, tooth staining usually occurs when dietary components are deposited or adsorbed on the teeth. Extrinsic staining can penetrate the tooth through the porosity of the enamel and be taken up by the dentin, resulting in tooth staining [2,43]. To further explore the bleaching effects of ZnPc(Lys)_5_ on the stained dentin blocks, we used a demineralized dentin staining model to simulate the stained teeth. Then, we directly compared the bleaching effects of different bleaching agents on stained dentin blocks. The dentin slices were bleached with deionized water, ZnPc(Lys)_5_, 10% HP, and 30% HP for up to 48 h after dyeing for 168 h (7 days). Table 1 displays the color parameters (L*, a*, b*, and W) of samples in four groups before bleaching. Before bleaching, no statistical difference was reported for the color parameters of each group.

**Figure 3:**
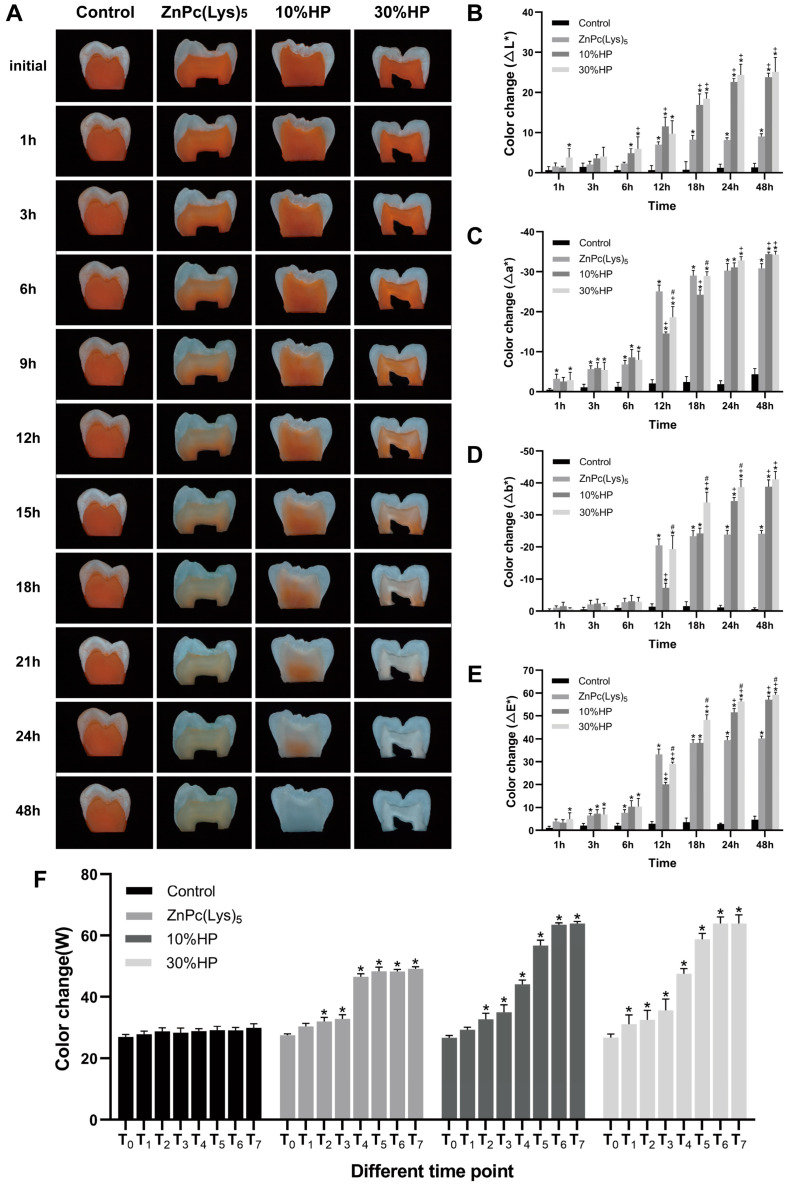
The bleaching effects of different solutions on dentin. (**A**) The bleaching effect as viewed through a stereoscopic microscope at different time points. (**B**–**E**) ∆L*, ∆a*, ∆b*, and ∆E* change in each group at different time points. Statistically significant differences between groups at different time points are indicated by * compared to the control group, + compared to the ZnPc(Lys)_5_ group, or # compared to the 10% HP group, respectively. (**F**) W value change in each group at different time points. T_0_, measurement before bleaching; T_1_, measurement after bleaching for 1 h; T_2_, measurement after bleaching for 3 h; T_3_, measurement after bleaching for 6 h; T_4_, measurement after bleaching for 12 h; T_5_, measurement after bleaching for 18 h; T_6_, measurement after bleaching for 24 h; T_7_, measurement after bleaching for 48 h. Statistically significant differences between different time points are indicated by * compared to T_0_.

Figure 3 shows the mean color changes of dentin blocks after bleaching at different time points. The absolute values of ∆L*, ∆a*, and ∆b* of the ZnPc(Lys)_5_, 10% HP, and 30% HP groups increased with time. Moreover, the color of dentin blocks moved towards white, green, and blue. The results of two-way ANOVA of ∆E*, ∆L*, ∆a*, ∆b*, and W values showed that the ∆E* values of the ZnPc(Lys)_5_, 10% HP, and 30% HP groups increased significantly after 3 h of bleaching compared to the control group (*p* < 0.05). In addition, the bleaching effects of the 10% HP and 30% HP groups were not as good as that of ZnPc(Lys)_5_ before the 12th hour of bleaching (*p* < 0.05). The color of the ZnPc(Lys)_5_ group changed significantly at 6 h, while the 10% HP and 30% HP groups changed clearly at 12 h and 9 h, respectively. Furthermore, the intra-group comparison showed similar results: the W values of the ZnPc(Lys)_5_, 10% HP, and 30% HP groups increased significantly (*p* < 0.05) in contrast to the control group, which did not change significantly (*p* > 0.1) with the extension of bleaching time. The abovementioned analyses confirmed that ZnPc(Lys)_5_, 10% HP, and 30% HP have good bleaching effects on dentin staining. Compared to the 10% HP group and 30% HP group, the ZnPc(Lys)_5_ group had a faster bleaching speed, and the clear color change occurred after 6 h of treatment, while the obvious color change occurred after 9 h in the 10% HP and 30% HP groups (Figure 3A). However, it is worth noting that, although ZnPc(Lys)_5_ showed a faster bleaching rate, its final bleaching effect was weaker than that of 10% HP and 30% HP. In addition, the dentin blocks treated with 30% HP showed excessive white areas and radial white stripes (Figure 3A). This was because 10% HP and 30% HP continued to bleach teeth after the color of Orange II degraded. Studies have shown that HP can cause changes in the organic and inorganic components of teeth [10,44]. In addition, the study conducted by Jiang et al. [45] showed that HP could bleach dentin by oxidizing dentin phosphoprotein. Therefore, it is speculated that HP changes the structure of dentin components and oxidizes dentin phosphoproteins so that HP can continue to bleach dentin after completely bleaching the Orange II. As a result, the final bleaching effects of HP are stronger than that of ZnPc(Lys)_5,_ with the appearance of excessive white areas and radial stripes in dentin blocks.

It should be pointed out that in vitro research cannot entirely replicate clinical practice; it remains a challenge to control all variables that arise in the human body, such as temperature, diet, and pH levels. These factors differ from person to person, making it difficult to standardize experiments. Moreover, the thickness of dentin can vary significantly between teeth and even within the same tooth, which can affect the effectiveness of bleaching treatments. Further clinical trials in humans are needed to translate this material into a product. The results obtained here will guide the design of human clinical trials.

### 2.4. The Restaining of Dentin Blocks after Tooth Bleaching

**Figure 4:**
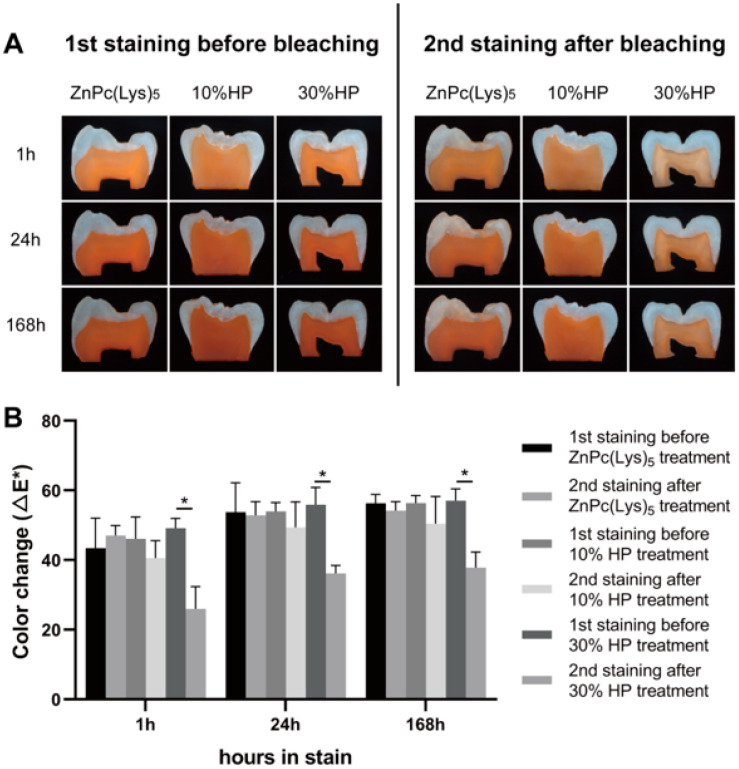
The restaining of dentin blocks. (**A**) Comparison of the effects of Orange II staining before and after bleaching for 48 h. (**B**) Mean color change (∆E*) of dentin samples stained by Orange II before and after bleaching for 48 h. A significant difference between the two groups is indicated by *.

The damage degree of collagen by different bleaching agents was indirectly judged by comparing the staining ability of the dentine block after bleaching with that before bleaching. After destaining with ZnPc(Lys)_5_, 10% HP, and 30% HP for 48 h, all of the dentin samples were restained with Orange II for 168 h (Figure 4A). The analysis of the ∆E* value (Figure 4B) shows that the secondary uptake of Orange II in the dentin blocks of the 30% HP group indicated a significant reduction compared to the equivalent sample following 168 h of the initial staining (*p* < 0.0001). Notably, there was no significant reduction in secondary Orange II uptake in the ZnPc(Lys)_5_ and 10% HP groups (*p* > 0.1). Researchers have studied the staining mechanism of dietary stains in dentin. After using surface plasmon resonance technology to measure the interactions between Orange II and different proteins of dentin in real time, they found that collagen type I had a moderate binding ability with Orange II, while the other protein components had a weak binding ability to Orange II. This suggests that collagen type I is closely related to dentin staining [40]. Therefore, via the evaluation of the recoloration degree of dentin blocks, it can be concluded indirectly that 10% HP and ZnPc(Lys)_5_ do not have a significant effect on collagen type I, while 30% HP damages collagen type I to a certain extent.

### 2.5. Physicochemical Properties of Post-Bleaching Dentin Surface

**Figure 5:**
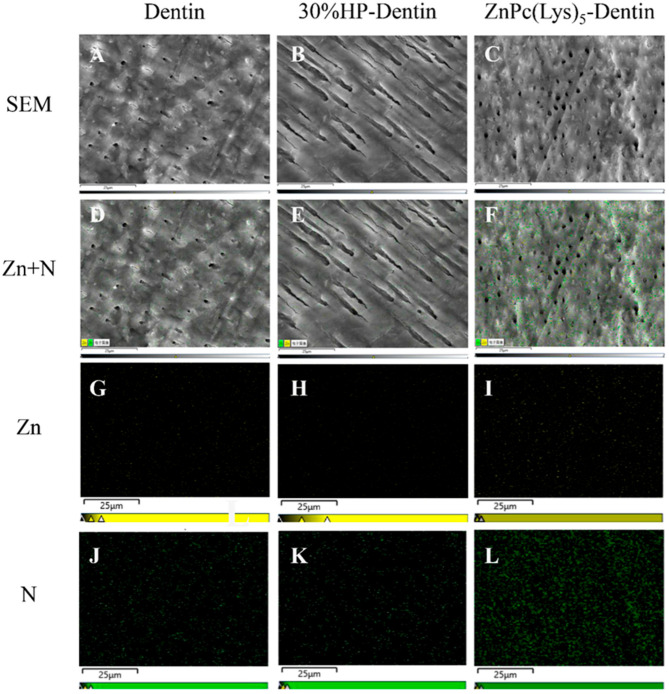
Dentin surface morphology (**A**,**B**) and elemental distribution (**D**–**L**). (**A**,**D**,**G**,**J**) are SEM images of untreated dentin. (**B**,**E**,**H**,**K**) are SEM images of 30%-HP-treated dentin. (**C**,**F**,**I**,**L**) are SEM images of ZnPc(Lys)_5_-treated dentin. (**G**,**H**,**I**) are scattered electron images of Zn (yellow). (**J**,**K**,**L**) are scattered electron images of N (green).

We analyzed the morphology of the dentin surfaces of different treatment groups by SEM. The surface morphology of dentin treated with ZnPc(Lys)_5_ (Figure 5A) was basically the same as that of blank dentin (Figure 5C), and both had a uniform distribution of pores, indicating that ZnPc(Lys)_5_ had no adverse effect on the dentin surface. In contrast, 30% HP (Figure 5B) changed the surface morphology with the complete loss of the regular and ordered pore structure. A small amount of ZnPc(Lys)_5_ was found to bind to the dentin surface in ZnPc(Lys)_5_-treated dentin using the XPS element scan (Figure 5D–F) and Zn (Figure 5G–I) and N (Figure 5J–L) imaging on the surface.

We also analyzed the elements of the dentin surface by EDS. The results indicated the presence of carbon, oxygen, phosphorus, and calcium in the dentin with P and Ca as main elements (Figure S1A). Upon treatment with 30% HP, the amount of C decreased (Figure S1B), reflecting the destruction of organic matter on the surface of the 30%-HP-treated dentin. In ZnPc(Lys)_5_-treated dentin, some forms of Zn were found, but P and Ca remained the main elements, clearly indicating that ZnPc(Lys)_5_ is bound to the dentin surface.

X-ray photoelectron spectroscopy (XPS), a surface analysis technique, was also used to characterize the surface elements of materials and their valency states. The sampling depth of the XPS technique is generally from 5 to 10 nm. The elements on the dentin surface were analyzed by XPS, which showed that 30%-HP-treated dentin samples and ZnPc(Lys)_5_-treated dentin samples both contained O, Ca, C, N, and P. In addition to these elements, the ZnPc(Lys)_5_-treated dentin samples also contained forms of Zn (Figure S2) with the peaks at 1021.68 eV and 1044.38 eV assigned to Zn 2p_3/2_ and Zn 2p_1/2_, respectively. This is consistent with the mapping data showing that the dentin surface contained some ZnPc(Lys)_5_. This is why the dentine mass appears slightly blue-green after bleaching. Studies have shown that the most important factor in self-perceived tooth whitening is the b* value [46], and the subjective response to the improvement of whiteness and patient satisfaction is significantly correlated with changes in the b* value [47]. Additionally, several studies have introduced the chemical blue covarine into whitening products to deposit blue covarine on the tooth surface, apply optical principles to change the b* value, change the tooth from a yellow to a blue tone, reduce the yellowness of teeth, and, thus, improve the overall perception of tooth whiteness [48,49,50]. Therefore, a little turquoise as produced in this study will increase the whiteness of teeth to some extent.

### 2.6. The Morphological Characterization of Dentin Collagen Fibers after Tooth Whitening

**Figure 6:**
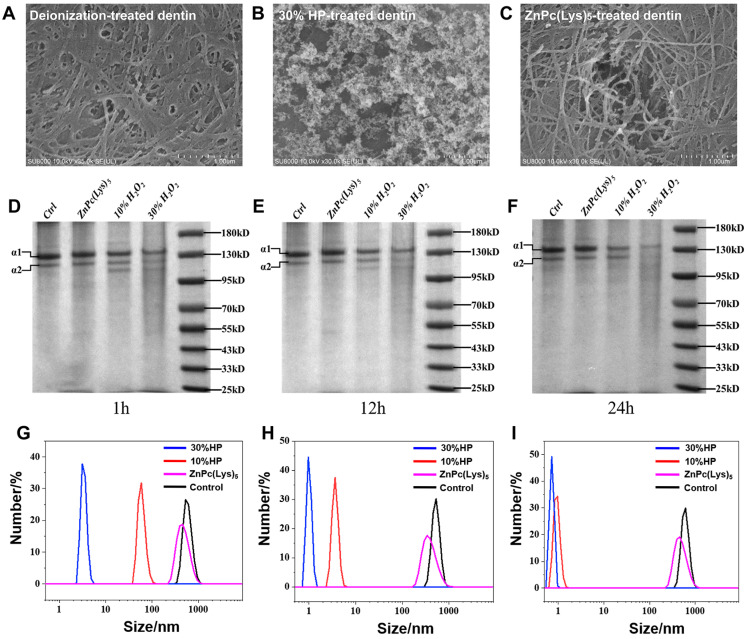
(**A**–**C**) Studies on the surface morphology of demineralized dentin collagen fibers. (**A**) is SEM image of deionization-treated dentin. (**B**) is SEM image of 30%-HP-treated dentin. (**C**) is SEM image of ZnPc(Lys)_5_-treated dentin. (**D**–**F**) Content of collagen type I after treatment with different solutions. Collagen type I after treatment for 1 h (**D**), 12 h (**E**), and 24 h (**F**). (**G**–**I**) The molecular size of collagen type I after treatment with different solutions. DLS characterization of 30% HP, 10% HP, and ZnPc(Lys)_5_ deionized water treatment for 1 h (**G**), 24 h (**H**), and 48 h (**I**).

Next, we analyzed the surface morphology of the demineralized dentin collagen fibers by SEM. In the control group of dentin, relatively intact collagen fibers were observed (Figure 6A). The 30% HP treatment led to the destruction of the collagen fiber meshwork, giving a rough surface (Figure 6B). In the photosensitizer-treated group, the collagen fibril meshwork was basically intact, with only a small amount of collagen fibril breakage (Figure 6C). The results showed that the photosensitizer did not significantly damage collagen type I, while HP significantly degraded collagen type I. The destruction of collagens by HP was consistent with the findings of previous studies. Alraies et al. [40] studied collagen type I by SDS-PAGE and silver staining, and the results showed that protein degradation occurred when 12% and 30% HP were combined with collagen type I. In addition, some researchers have analyzed the influence of HP on dentine collagen and other organic matter by Raman spectroscopy and found that HP degrades collagen [20,51]. Toledano et al. [22] used radioimmunoassay to evaluate the effects of HP on collagen type I by measuring the carboxyl terminal peptide content of collagen type I. The results showed that HP could significantly degrade the collagen. The different action mechanisms of the photosensitizer and HP could be the reason for this observation. HP decomposition produces free radicals that bleach the chromophore and, at the same time, damage the collagen in dentin [22]. Photodynamic therapy refers to the energy transfer of photosensitizers via types I and II reaction mechanisms to produce reactive oxygen species (ROS) under the excitation of a specific wavelength light source. The ROS will react with the chromophore to bleach the tooth. A key requirement for the ROS generation with the photosensitizer is the light illumination. The ROS will be extinguished rapidly upon removal of the light illumination. The typical light penetration depth is 5.0–8.0 mm in human tissue for the photosensitizer used in this work [52]. For the dentin, the LED light is quite unlikely to penetrate much. Thus, the ROS generated by the PDT will have a gradient distribution with more ROS on the surface and much less inside the dentin structure. This action mode of photodynamic therapy is different from that of the hydroxyl radical produced by HP, which can easily penetrate deep into dentin.

### 2.7. The In Vitro Effect of Bleaching Agents on Collagen Type I

In order to observe the effects of different agents on collagen type I more directly, SDS-PAGE and DLS were used to analyze the protein destruction of different bleaching agents directly interacting with purified collagen type I for a certain time. The purified human collagen type I was examined via SDS-PAGE. Figure 6D–F show the electrophoretic results of collagen type I after the application of bleaching agents. There is a clear protein band at the molecular weights of 130 kD and 110 kD, which indicate the α 1 and α 2 chains of collagen type I, respectively. It was observed that collagen type I was not degraded after treatment with the photosensitizer for up to 24 h, while treatment with 10% HP and 30% HP at 1 h, 12 h, and 24 h resulted in the degradation of collagen type I. Compared to 10% HP, treatment with 30% HP led to increased degradation.

We also measured the molecular size of human-derived collagen type I with different treatment based on dynamic light scattering (DLS). Figure 6G–I show treatment with 30%HP, 10%HP, the photosensitizer, and deionized water for 1 h (A), 24 h (B), and 48 h (C). The results showed that the treatment with HP (10% or 30%) destroyed collagen after 1 h of incubation, reducing the molecular size of collagen to less than 100 nm. With the increase in incubation time, the destruction of collagen by 30% HP or 10% HP was more significant. At 48 h, the size of collagen decreased to about 1 nm. In contrast, the molecular size of collagen in the photosensitizer-treated group remained basically constant after 1 h, 24 h, and 48 h of treatment, which was basically the same as that of the deionized water group. These data show that the photosensitizer does not destroy collagen. The results of SDS-PAGE and DLS both showed that ZnPc(Lys)_5_, as a new bleaching agent, did not significantly damage collagen type I, while 10% HP and 30% HP degraded collagen type I. This is in agreement with the results of the SEM data that showed that ZnPc(Lys)_5_ did not significantly damage collagen fibril meshwork, while HP significantly degraded collagen fibril meshwork. These results confirm that ZnPc(Lys)_5_-mediated photodynamic therapy for tooth bleaching does not significantly damage the collagen type I of dentin.

### 2.8. Detection of Singlet Oxygen Production during PDT for Bleaching

Photodynamic therapy typically generates two types of ROS under the excitation of a specific wavelength light source. In the type I reaction mechanism, the photosensitizer directly combines with the substrate or solvent to generate superoxide and hydroxyl radicals, while the photosensitizer in type II reacts directly with oxygen molecules, resulting in energy transfer and singlet oxygen production [53,54,55]. Previous studies have shown that type II reaction is the main mechanism of the photosensitizer used here. In order to confirm whether singlet oxygen (^1^O_2_) produced by type II reaction plays a bleaching role, the Singlet Oxygen Sensor Green (SOSG) fluorescence probe experiment was used to assess the change in ^1^O_2_ content in this study. Figure S3 shows the experimental results of the SOSG fluorescence probe. After ZnPc(Lys)_5_ was irradiated by the LED light source in Orange II solution, the fluorescence intensity of SOSG in the solution increased with time, indicating that ZnPc(Lys)_5_ could produce ^1^O_2_ under LED light source irradiation, and there was a type II photodynamic reaction. Under the conditions of light avoidance, the fluorescence intensity of SOSG in ZnPc(Lys)_5_ and the Orange II solution did not change significantly with the increase in time. These results confirm that ZnPc(Lys)_5_-mediated photodynamic therapy occurs via a type II response in aqueous Orange II. Pentapylysine zinc phthalocyanine is a water-soluble cation photosensitizer. Previous studies have shown that its singlet oxygen yield in DMSO solution is 0.64 ± 0.02 [56], which is similar to that of zinc phthalocyanine in DMSO (0.67) [57]. This study further confirmed that ZnPc(Lys)_5_-mediated photodynamic therapy plays a bleaching role via singlet oxygen produced by the type II reaction mechanism.

## 3. Materials and Methods

### 3.1. Preparation of ZnPc(Lys)_5_ Photosensitizer

**Figure 7:**
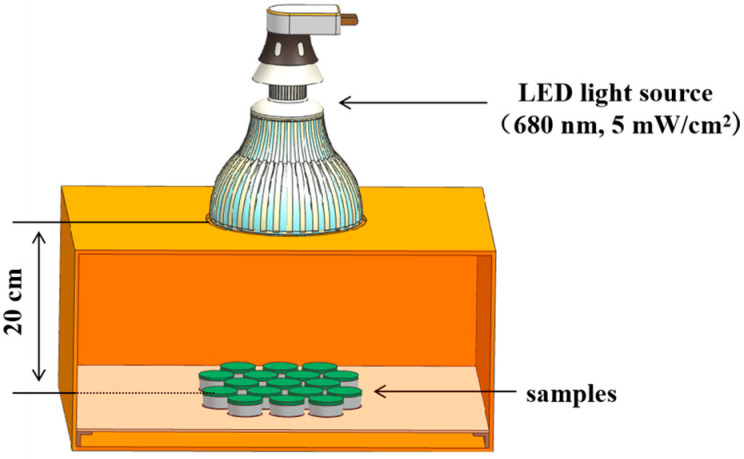
Experiment diagram for bleaching.

The photosensitizer ZnPc(Lys)_5_ for this study was synthesized to high purity by our team from Fuzhou University as previously reported [33]. The structural formula is shown in Figure S4. Chemical Formula: C_63_H_76_N_18_O_7_Zn. The UV/Vis absorption spectrum of ZnPc-(Lys)_5_ in DMSO is typical for ZnPc, with the strongest absorption at 678 nm (ε = 118,380 L mol^−1^cm^−1^) [57]. LED light source (Sanyi Illumination, Inc., Nanning, China, 680 nm, 5 mW/cm^2^) was used in this study and illuminated on samples at the distance of 20 cm from their surface (Figure 7).

### 3.2. Discoloration of Orange II by ZnPc(Lys)_5_ Photosensitizer and HP in Solution

The Orange II dye was used to evaluate the bleaching efficacy of different bleaching agents in Eppendorf tubes. The experiment was divided into two groups: one group was exposed to the indoor fluorescent lamp, and the other group was exposed to an LED light source of 680 nm with a power of 5 mW/cm^2^. In each group, deionized water, 10% HP, 30% HP, and 25 μM ZnPc(Lys)_5_ solution was mixed with Orange II (0.15 mM) solution of the same volume.

Eight groups (Groups A to H) were established in the Eppendorf tubes:

- Group A: 200 μL of Orange II were reacted with 200 μL of deionized water under an indoor fluorescent lamp.
- Group B: 200 μL of Orange II were reacted with 200 μL of 10% HP under an indoor fluorescent lamp.
- Group C: 200 μL of Orange II were reacted with 200 μL of 30% HP under an indoor fluorescent lamp.
- Group D: 200 μL of Orange II were reacted with 200 μL of ZnPc(Lys)_5_ under an indoor fluorescent lamp.
- Group E: 200 μL of Orange II were reacted with 200 μL of deionized water under a 680 nm LED lamp.
- Group F: 200 μL of Orange II were reacted with 200 μL of 10% HP under a 680 nm LED lamp.
- Group G: 200 μL of Orange II were reacted with 200 μL of 30% HP under a 680 nm LED lamp.
- Group H: 200 μL of Orange II were reacted with 200 μL of ZnPc(Lys)_5_ under a 680 nm LED lamp.

To compare the concentration of Orange II, we measured the absorbance of Orange II (484 nm) using a microplate reader (BIO-RADiMark, Bio-Rad, Hercules, CA, USA) at hourly intervals. The efficiency of bleaching agents was determined by the decrease in absorbance of Orange II. At the same time, ZnPc(Lys)_5_ solution without Orange II was added under the conditions of LED lamp and indoor fluorescent lamp, respectively, and the Eppendorf tubes were photographed at 0.5, 3, and 6 h.

### 3.3. Sample Preparation

Patient consent and ethical approval from the Ethics Committee of the School and Hospital of Stomatology, Fujian Medical University (reference number: FMUSS-20-045) were obtained for the collection of human teeth. Dental integrity was required, and crowns with caries, fluorosis, and tetracycline teeth were excluded. Following the removal of periodontal ligaments, calculus, and dirt, the teeth were cleaned with ultrasound for 20 min and then preserved in 0.1% thymol crystals to inhibit bacterial growth. A high-speed handpiece (Pana-Max2, NSK, Tokyo, Japan) was used to amputate the collected teeth at the cemento–enamel junction under water cooling. The crown was cut along the proximal and distal direction of the teeth into longitudinal sections of dentin with a thickness of 0.5 mm. All samples were polished using 600, 800, and 1200 grit silicon carbide paper with water irrigation and washed for 20 min by ultrasonic washing.

### 3.4. Sample Treatments and Color Evaluation

Ten dentin sections, prepared from five permanent molars, were randomly divided into the demineralization group and the control group (n = 5). Each section was placed in one well of a 12-well plate, treated with either demineralizing solution (10% EDTA, pH 7.2) or phosphate-buffered saline (PBS) for 3 days, and then stained with Orange II (0.15 mM). The intensity of dye uptake was quantified at 0, 1, 12, 24, 48, 72, and 168 h. A digital camera attached to a stereo microscope (Zeiss, stemi 508) was used to record the color changes of dentin slices at low magnification (×0.63) under an automatic ring-light electronic flash illumination with the same parameters (exposure time: 14 s, white balance: 5500 K) to achieve standard conditions. The same apparatus was used for the process of tooth stain development, bleaching, and restaining. Three non-reflective points were selected for color measurement of each dentin sample. Subsequently, image processing software (Photoshop 13.0, Adobe System Inc., San Jose, CA, USA) was used to transform the dentin section images into L*, a*, and b* values according to the International Commission on Illumination (CIE) Lab system. The Lab system evaluates the color changes of dentin blocks using a three-dimensional space. The L* value indicates the brightness of the sample and ranges from 0 (black) to 100 (white). The a* value represents the degree of green (−) or red (+), and the b* value represents the degree of blue (−) or yellow (+). The calculation formulas (∆E*, Whiteness (W)) of the entire color change are as follows:

∆E* = [(L_i_* − L_0_*)^2^ + (a_i_* − a_0_*)^2^ + (b_i_* − b_0_*)^2^]^1/2^, (1)

W = 100 − [(100 − L_i_*)^2^ + a_i_*^2^ + b_i_*^2^]^1/2^ (2)

Each time point is represented by the subscript “i”, and the baseline is represented by the subscript “0”.

After staining, demineralized dentin sections were randomly treated with deionized water, 10% HP, 30% HP, and ZnPc(Lys)_5_ for 48 h (n = 5). All groups were exposed to an LED light source of 680 nm with a power of 5 mW/cm^2^. The digital image analysis described above was used to monitor the color changes of dentin slices at 0, 1, 3, 6, 12, 18, 24, and 48 h. Then, dentin blocks that had been bleached were restained with Orange II and their stain intensity was measured at 1, 24, and 168 h, as described above.

### 3.5. SEM Characterization of Dentin Sections and Collagen Fibers

Dentin sections were placed in 12-well plates and soaked in 2 mL of either deionized water, 30% HP, or ZnPc(Lys)_5_ (25 μM) for 48 h. The ZnPc(Lys)_5_ group was given additional lighting at 680 nm with a light dose of 162 J/cm^2^. After soaking, the sections were rinsed three times with deionized water, followed by acidification with 37% phosphoric acid for 1 min and washing again with water three times. Next, the sections were immersed in sterile secondary water containing 2.5% (*v*/*v*) glutaraldehyde for fixation and stored overnight at 4 °C. Finally, the samples were dehydrated sequentially with ethanol at 30%, 50%, 70%, 90%, and 100% (*v*/*v*) concentrations, followed by drying at room temperature. The dried samples were fixed on the sample table with conductive adhesive and electroplated with gold for 1 min. Scanning electron microscopy (SEM: New Generation SU8010, Hitachi, Newton Aycliffe, Btitain) was used to characterize the morphology of dentin collagen fibers for energy dispersive spectroscopy (EDS).

### 3.6. X-ray Photoelectron Spectroscopy of Dentin Sections

Dentin was also treated with 30% HP or ZnPc(Lys)_5_ (25 μM) for 48 h. The ZnPc(Lys)_5_ group was additionally illuminated at 680 nm to a light dose of 162 J/cm^2^, followed by drying naturally at room temperature. The samples were tested by fixing the dentin to the sample testing table with conductive adhesive. The sample was placed into the analysis chamber at a pressure of less than 2.0 × 10^−7^ mbar. The X-ray source was Al Kα rays (hv = 1486.6 eV) with a spot size of 400 μm; vacuumed to better than 5.0 × 10^−7^ mBar; and had an operating voltage of 12 kV, a filament current of 6 mA, a full spectrum scan energy of 150 eV in steps of 1 eV, and a narrow spectrum scan energy of 50 eV in steps of 0.1 eV. The instrument model is Scientific K-Alpha, Thermo, Waltham, MA, USA.

### 3.7. Effects of Bleaching Agents on Collagen I

In this study, collagen type I (C7774, Sigma, Cibolo, TX, USA) has been dissolved in 0.5 M acetic acid at 3 mg/mL and then mixed with deionized water, 25 μM of ZnPc(Lys)_5_, 10% HP, and 30% HP for 1, 12, and 24 h at room temperature. All groups were exposed to an LED light source of 680 nm with a power of 5 mW/cm^2^. Samples were mixed with loading buffer (P0015F, Beyotime, Shanghai, China) and boiled for 3–5 min to denature the protein. Next, 15 μL samples were split by SDS-PAGE using 10% Protein Precast gels (F15010LGel, ACE Biotechnology, Nanjing, China), 0.05 M Tris, 0.05 M Mops, and 0.1% SDS running buffer at 180 v for 15 min. The gels were stained with Coomassie brilliant blue (P0017F, Beyotime, Shanghai, China) following the manufacturer’s instructions and were scanned using the BioDoc-It^TM^ Imaging System (BIODOC-IT-220, UVP, Greenwood, IN, USA).

### 3.8. Dynamic Light Scattering (DLS) Measurement of Collagen I

Human-derived collagen I was dissolved in 0.5 M acetic acid and mixed with 30% HP (990 µL), 10% HP, or ZnPc(Lys)_5_ (25 µM, 1 mg/mL, 10 µL, respectively). The ZnPc(Lys)_5_ group was further illuminated (680 nm) to a light dose of 162 J/cm^2^. The blank control group was a mixture of 0.5 M acetic acid solution (990 µL) and 1 mg/mL (10 µL) of collagen. The effects of different treatments on collagen particle size were measured after 1 h, 24 h, and 48 h incubation on Nano ZS-90 instrument.

### 3.9. Detection of Singlet Oxygen with SOSG

According to the instructions of the manufacturer, 100 μg of Singlet Oxygen Sensor Green (SOSG, Invitrogen, Carlsbad, CA, USA) powder was dissolved in a 33 μL methanol solution to prepare a 5 mM reserve solution. The reserve solution was stored at −20 °C and prepared for the working liquid 30 min before the formal experiment. Under the conditions of light avoidance, 25 μM of ZnPc(Lys)_5_ was mixed with 0.15 mM Orange II solution in equal volume and added to the SOSG solution with a final concentration of 1 μM. One group was irradiated vertically with the 680 nm LED lamp to make its irradiance 5 mW/cm^2^, and the other group was placed in a dark room (n = 3). According to the operating instructions, the excitation wavelength of SOSG is 495 nm with an emission wavelength of 535 nm, and the fluorescence emitted by SOSG is detected by a microplate reader (BIO-RADiMark, Bio-Rad, Hercules, CA, USA) every 20 s.

### 3.10. Statistical Analyses

With different groups and time as factors, the overall differences in the ∆L*, ∆a*, ∆b*, ∆E*, and W scores were analyzed using two-way repeated measures analysis of variance (ANOVA) to determine statistically relevant differences between the deionized water, ZnPc(Lys)_5_, 10% HP, and 30% HP groups. The differences in ∆L*, ∆a*, ∆b*, ∆E*, and W scores at each evaluation time point were tested by one-way factorial ANOVA followed by the Bonferroni post hoc test. The value of *p* < 0.05 was considered significant. Statistical analyses were performed using GraphPad Prism9 (GraphPad Software, La Jolla, CA, USA).

## 4. Conclusions

We demonstrate here that ZnPc(Lys)_5_-mediated photodynamic effect has satisfactory and fast bleaching efficacy, when compared to hydrogen peroxide, on dentin staining without any discernable detrimental effects on dentin and its embedded protein collagen type I, the main organic component of dentin. Although the final bleaching effect of this material is slightly lower than that of hydrogen peroxide, it does not damage the dentin collagen, unlike hydrogen peroxide, which can reduce the mechanical properties of the teeth due to protein destruction. In addition, the pale bluish color of phthalocyanine is favored in dental practice. Together with our previous work of the ZnPc(Lys)_5_-mediated bleaching of tooth enamel, we clearly demonstrate that the phthalocyanine-type photosensitizer has great promise to be used in dental practice.

## Figures and Tables

**Table 1:** Color parameters of samples in each group before bleaching. Data are presented as mean ± standard deviation (*n* = 5).

	Group			
	Control	ZnPc(Lys)_5_	10% HP	30% HP
L*	40.80 ± 0.93	41.06 ± 0.49	41.06 ± 1.32	40.47 ± 1.07
a*	28.13 ± 0.83	26.33 ± 1.13	27.80 ± 1.04	26.93 ± 0.98
b*	32.20 ± 1.72	32.93 ± 0.64	33.60 ± 1.30	33.00 ± 1.45
W	26.95 ± 0.83	27.52 ± 0.45	26.67 ± 0.74	26.78 ± 1.09

## Data Availability

The data presented in this study are available on request from the corresponding authors.

## Supplementary Materials

The following supporting information can be downloaded at: https://www.mdpi.com/article/10.3390/molecules28104223/s1. Figure S1: Distribution of energy dispersive spectroscopy (EDS) in different solution treatment groups. (A) Dentin; (B) 30% HP-treated dentin; (C) ZnPc(Lys)_5_-treated dentin. Figure S2: XPS characterization. (A) XPS of 30% HP-treated dentin; (B) XPS of ZnPc(Lys)_5_-treated dentin; (C) XPS of Zn 2p energy level orbitals. Figure S3: The detection of ^1^O_2_ produced by the SOSG probe test. Figure S4: The chemical structure of ZnPc(Lys)_5_.

## Sample Availability

Samples of the compound pentalysine β-carbonyl-phthalocyanine zinc (ZnPc(Lys)_5_) are available from the authors.
