# Supplementary material for: Dental Bleaching with Phthalocyanine Photosensitizers: Effects on Dentin Color and Collagen Content
Source: Molecules. 2023 May 22;28(10):4223. doi: 10.3390/molecules28104223 (PMC10224055; doi:10.3390/molecules28104223)
Supplement: Supplementary file 1 [file molecules-28-04223-s001.zip › molecules-2296468-supplementary.pdf]

Supporting information for

DDental Bleaching with Phthalocyanine Photosensitizers: Effects on Dentin Color and Collagen Content

Zhouyan Wu [1,2,†], Guodong Wang [3,†], Zhiming Li [1], Zhengquan Li [1], Dandan Huang [1], Mingdong Huang [3,*] and Minkui Lin [1,4,*]

1 Fujian Key Laboratory of Oral Diseases & Fujian Provincial Engineering Research Center of Oral Biomaterial & Stomatological Key Lab of Fujian College and University, School and Hospital of Stomatology, Fujian Medical University, 246 Yangqiao Zhong Road, Fuzhou 350002, China; 15659036770@163.com (Z.W.)

2 The Second Affiliated Hospital of Fujian University of Traditional Chinese Medicine, 282 Wusi Road, Fuzhou 350003, China

3 College of Chemistry, Fuzhou University, 2 Xueyuan Road, Fuzhou 350108, China

4 Institute of Stomatology & Laboratory of Oral Tissue Engineering, School and Hospital of Stomatology, Fu-jian Medical University, Fuzhou 350002, China

* Correspondence: linmk105@sina.com (M.L.); HMD_lab@fzu.edu.cn (M.H.).

† These authors contributed equally to this work

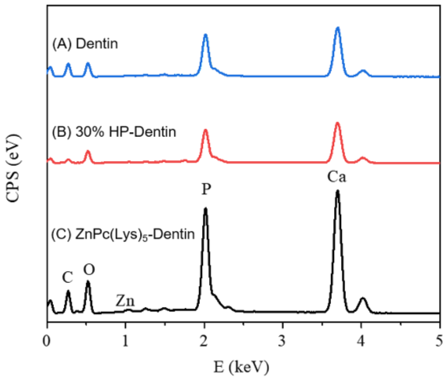

Figure S1. Distribution of energy dispersive spectroscopy (EDS) in different solution treatment groups. (A) Dentin (B) 30% HP-treated dentin (C) ZnPc(Lys)$_5$-treated dentin.

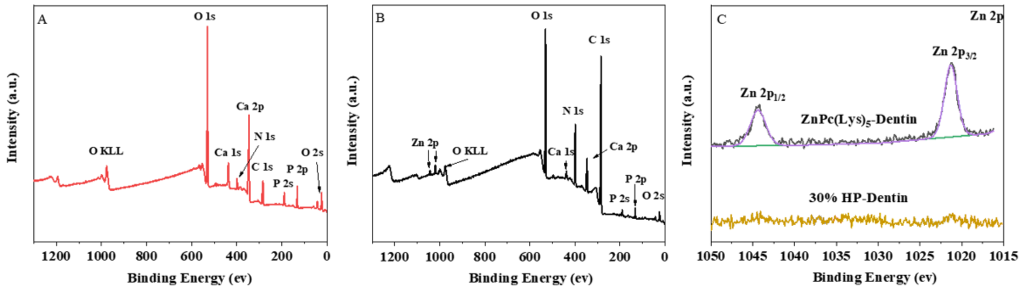

Figure S2. XPS characterization (A) XPS of 30% HP-treated dentin (B) XPS of ZnPc(Lys)$_5$-treated dentin (C) XPS of Zn 2p energy level orbitals.

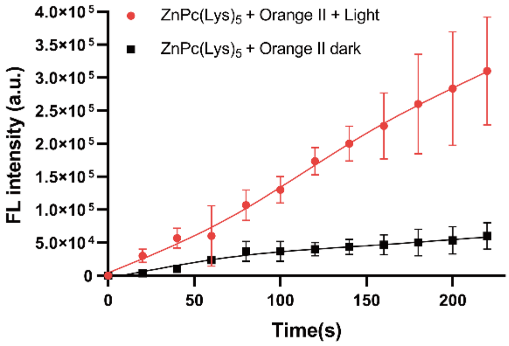

Figure S3. The detection of $^1O_2$ produced by the SOSG probe test.

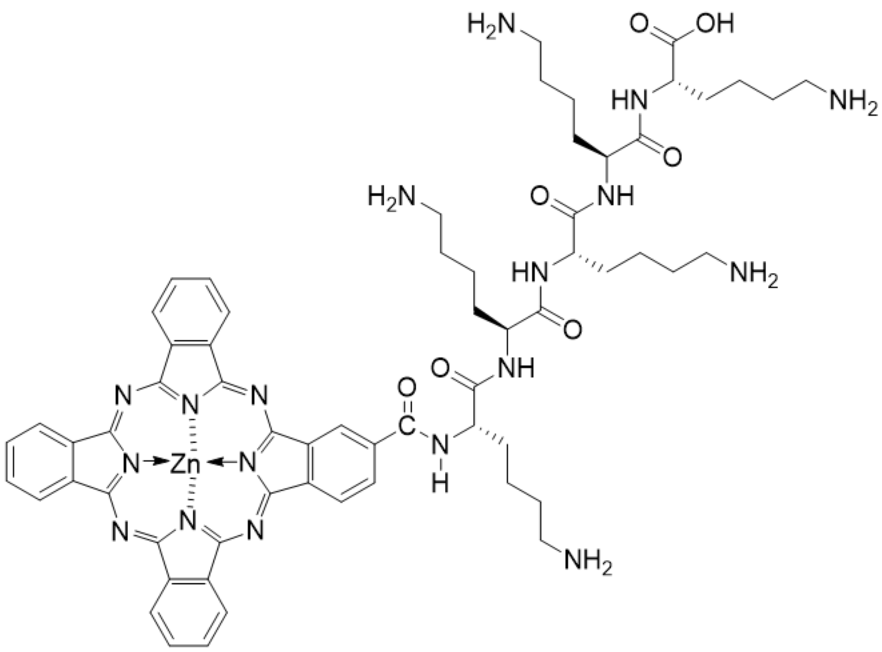

Figure S4. The chemical structure of ZnPc(Lys)$_5$.
